# Detecting Synchronous Parathyroid Adenoma and False-Positive Findings on Technetium-99m MIBI Single Photon-Emission Computed Tomography/Computed Tomography

**DOI:** 10.3390/diagnostics9020057

**Published:** 2019-06-01

**Authors:** Ji Young Lee, Hee-Sung Song, Jae Hyuck Choi, Chang Lim Hyun, Sang Ah Lee, Joon-Hyouk Choi, Seokjae Lee

**Affiliations:** 1 Department of Nuclear Medicine, Jeju National University Hospital, Jeju National University School of Medicine, Jeju-si 63241, Korea; easy02000@naver.com; 2 Department of Surgery, Jeju National University Hospital, Jeju National University School of Medicine, Jeju-si 63241, Korea; basson@hanmail.net; 3 Department of Pathology, Jeju National University Hospital, Jeju National University School of Medicine, Jeju-si 63241, Korea; venisua@jejunu.ac.kr; 4 Department of Endocrinology and Metabolism, Jeju National University Hospital, Jeju National University School of Medicine, Jeju-si 63241, Korea; sahe7@hanmail.net; 5 Department of Internal Medicine, Jeju National University Hospital, Jeju National University School of Medicine, Jeju-si 63241, Korea; valgom@hanmail.net; 6 Jeju National University College of Medicine and Graduate School of Medicine, Jeju-si 63241, Korea; Lsjtop@gmail.com

**Keywords:** parathyroid adenoma, Tc-99m MIBI scintigraphy, primary hyperparathyroidism, Tc-99m MIBI SPECT/CT, false-positive finding

## Abstract

Technetium (Tc)-99m-methoxyisobutylisonitrile (MIBI) single photon-emission computed tomography/computed tomography (SPECT/CT) is now being used increasingly for preoperative localization of parathyroid adenomas. Tc-99m-MIBI scintigraphy in a 52-year-old man with a diagnosis of primary hyperparathyroidism revealed two focal areas with retention of radioactivity in the left lobe of the thyroid gland on the delayed phase of MIBI SPECT/CT but no significant focal radioactive uptake on MIBI planar images. The patient subsequently underwent left partial parathyroidectomy. Histological analysis identified one lesion to be thyroid hyperplasia and the other to be parathyroid adenoma. This case demonstrates the value of MIBI SPECT/CT for localization of a parathyroid lesion when compared with planar images and that false-positive findings can lead to misdiagnosis in a patient with coexisting thyroid disease. An appropriate diagnostic work-up that includes Tc-99m MIBI SPECT/CT in addition to ultrasonography is helpful for an accurate diagnosis in patients with concomitant thyroid disease.

## 1. Introduction

Technetium-99m-mexhoxyisobutylisonitrile (MIBI) dual-phase scintigraphy is an imaging technique used in patients with primary hyperparathyroidism [1,2], an endocrine disorder in which one or more of the parathyroid glands are hyperfunctioning [3]. The cause of primary hyperparathyroidism in most cases is a single parathyroid adenoma, followed by multiple adenomas and hyperplasia [4]. Tc-99m MIBI is sequestered in the mitochondria of cells in parathyroid adenoma and hyperplasia. Therefore, scintigraphy allows preoperative localization of an abnormal parathyroid gland and minimally invasive surgical dissection [5].

Advances in hybrid imaging with single photon-emission computed tomography/computed tomography (SPECT/CT) have significantly increased the diagnostic potential of imaging in recent years. SPECT/CT can allow accurate localization, exclusion of physiological uptake, and detection of additional lesions because the CT component identifies anatomical landmarks and has higher resolution than that of SPECT [6,7]. Therefore, Tc-99m MIBI SPECT/CT has higher sensitivity than planar images and SPECT [8,9,10].

Herein, we report the unusual case of a patient with coexisting parathyroid adenoma and thyroid hyperplasia that were localized using Tc-99m MIBI SPECT/CT.

## 2. Case Report

**Figure 1:**
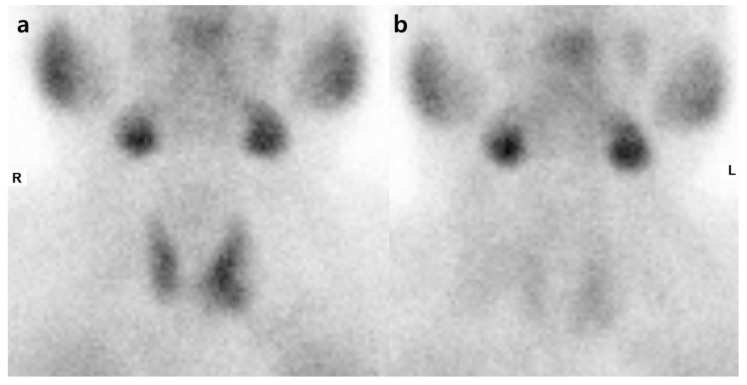
Dual-phase Tc-99m MIBI planar scintigraphy. The early images (**a**) show homogenous radioactive uptake in both lobes of the thyroid (enlarged left lobe) and the delayed images (**b**) show washout of radioactivity from the thyroid gland with no abnormal retention. R: right; L: left.

**Figure 2:**
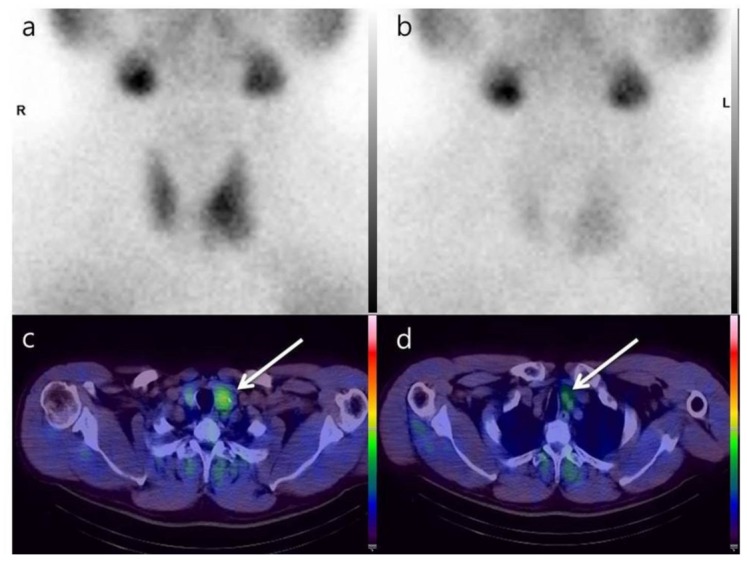
Parathyroid lesions detected by Tc-99m MIBI single photon-emission computed tomography/computed tomography. Two lesions (white arrows) with focal radioactive uptake were detected in the left thyroid lobe (**c**,**d**). These lesions were not detected by early (**a**) or delayed (**b**) Tc-99m MIBI planar scintigraphy images. R: right; L: left.

**Figure 3:**
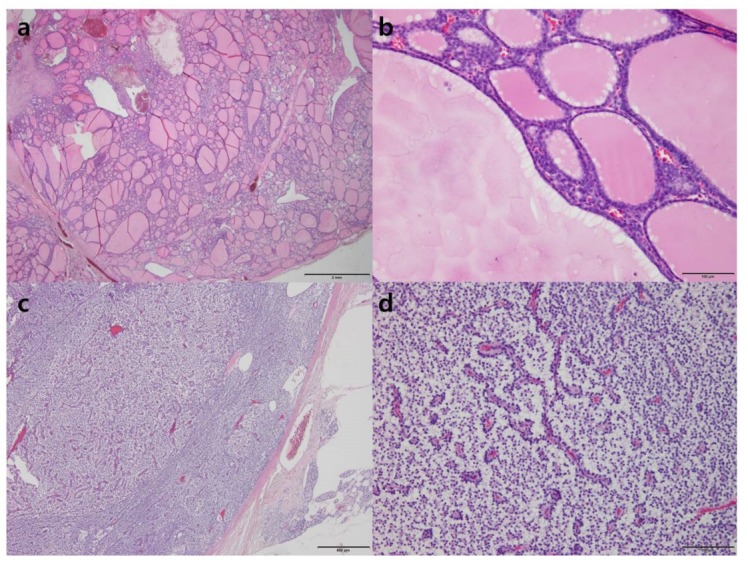
Pathologic sections of nodular hyperplasia of the thyroid gland (**a**,**b**) and parathyroid adenoma (**c**,**d**) with hematoxylin and eosin staining. Low-power (**a**, ×10) and high-power (**b**, ×200) microscopic views of the lesion show variably sized dilated follicles. The parathyroid adenoma (**c**, ×40, **d**, ×200) is composed mainly of chief cells.

A 52-year-old man with a history of hyperparathyroidism was referred to the nuclear medicine department for Tc-99m MIBI scintigraphy. A year earlier, he had been diagnosed with primary hyperparathyroidism on the basis of hypercalcemia (12.6 mg/dL; reference range, 8.6–10.1 mg/dL) and an increased parathyroid hormone level (98.1 pg/mL; reference range, 15–65 pg/mL). Previous ultrasonography (USG) of the neck had revealed a small nodular lesion in the left lobe of the thyroid gland that was suspected to be parathyroid adenoma/hyperplasia. A dual-phase Tc-99m MIBI scintigraphy examination was performed to investigate the parathyroid lesion, the first phase at 15 min after injecting 30 mCi of Tc-99m MIBI for a duration of 10 min and the second at 120 min for a duration of 10 min. However, no focal MIBI uptake was observed on delayed Tc-99m MIBI planar scintigraphy images (Figure 1). A year later, the patient revisited our department because of a steadily increasing serum parathyroid hormone level (120.1 pg/mL). There was still no significant focal radioactive uptake on early or late Tc-99m MIBI planar images. A Tc-99m MIBI SPECT/CT gamma camera (Symbia T6, Siemens Healthcare, Erlangen, Germany) was used to obtain the images of the neck and chest at 150 min after 30 mCi of Tc-99m MIBI injection for 15 min in the delayed phase. The images revealed two focal areas with retention of radioactivity in the left lobe of the thyroid. One lesion was observed in the lower portion of the left lobe and the other below the lower pole of the left lobe (Figure 2). We reported these two lesions as possible parathyroid adenoma or hyperplasia. The patient underwent left partial parathyroidectomy. The lesion in the lower portion of the left lobe was pathologically confirmed to be thyroid hyperplasia and the other lesion below the lower pole of the left lobe to be parathyroid adenoma (Figure 3). After excision of the parathyroid lesion, the serum parathyroid hormone level returned to normal (30.3 pg/mL). The authors have obtained the patient’s informed consent.

## 3. Discussion

Hyperparathyroidism is a common endocrine disorder that results in hypercalcemia, an elevated serum parathyroid hormone level, and hypophosphatemia. In its later stages, hyperparathyroidism may affect multiple organs, including the kidneys and bones. The cause of hyperparathyroidism is parathyroid adenoma in over 80% of cases, followed by parathyroid hyperplasia in 6–10% [4]. Surgical removal is the only curative treatment for parathyroid adenoma or hyperplasia. The most recently developed surgical approach involves unilateral cervical exploration to minimize the operating time and the risk of complications [5,11,12].

A sensitive and cost-effective modality for preoperative localization of a hyperfunctioning parathyroid lesion is essential for minimally invasive surgery. The current standard preoperative imaging used in primary hyperparathyroidism is USG of the neck and a Tc-99m MIBI planar scan. In recent years, MIBI SPECT/CT has been recognized as the most sensitive modality for acquiring anatomical information from CT and has improved the detection and localization of abnormal parathyroid lesions, whereas planar MIBI imaging has the limitation of a low sensitivity of approximately 70% [13]. SPECT/CT in particular can detect a deep-seated or ectopic parathyroid lesion that would not be found on USG or planar images with a sensitivity of around 90% [5,14,15,16,17]. Our case underscores the value of Tc-99m MIBI SPECT/CT for detection of lesions when compared with Tc-99m planar MIBI scintigraphy. 

Small-sized parathyroid lesions with low mitochondrial content have been shown to produce false-negative MIBI results. A previous study reported that the advantage of SPECT/CT over SPECT alone is most apparent for small adenomas weighing less than 210 mg [18]. Our patient also had a small-sized lesion that measured approximately 1 cm × 1cm in size and a chief cell-dominant parathyroid adenoma. Close observation on SPECT/CT with neck USG may reduce the number of false-negative findings for small parathyroid lesions. Combination of USG and SPECT/CT has been shown to increase the overall sensitivity for localization of parathyroid lesions to up to 95% [5]. 

MIBI is taken up by the mitochondria in thyroid and parathyroid tissue. Hyperfunctioning parathyroid tissues contain mitochondria-rich oxyphil cells and retain radioactivity for longer than the neighboring thyroid tissue [5,19]. Any mitochondria-rich cells may take up MIBI, so false-positive findings are possible. The most frequent entity with false-positive findings on a Tc-99m MIBI scan is a solid nodule such as thyroid adenoma, carcinoma, or lymphoma [19,20]. Similarly, in our case, thyroid hyperplasia was a false-positive finding on Tc-99m MIBI SPECT/CT. Tc-99m MIBI SPECT/CT may be more useful for localizing a parathyroid lesion than planar images, and false-positive findings on SPECT/CT can lead to misdiagnosis in a patient with coexisting thyroid disease. Concomitant thyroid nodules are common, occurring in 20–75% of cases in endemic areas. Therefore, the possibility of the coexistence of false-positive findings owing to thyroid lesions must be considered and further evaluation is required when another focal lesion is seen on a Tc-99m MIBI SPECT scan [21,22]. Furthermore, similar to this MIBI SPECT/CT, a recent study reported the importance of hybrid imaging using tumor-specific biomarkers in head and neck tumors, and this is thought to help the patients’ treatment guidance [23].

## 4. Conclusions

We encountered a rare case of synchronous parathyroid adenoma and thyroid hyperplasia that were localized using Tc-99m MIBI SPECT/CT. Tc-99m MIBI SPECT/CT is more useful for detecting and localizing small-sized parathyroid lesions than planar scintigraphy. However, SPECT/CT is often misleading in patients with coexisting thyroid disease. An appropriate diagnostic work-up that includes a combination of USG and Tc-99m MIBI SPECT/CT with careful interpretation is helpful for an accurate diagnosis in patients with concomitant thyroid disease.
